# Growth Differentiation Factor 15 (GDF-15) as a modulator of hepatic steatosis and fibrosis: insights from a 6-year retrospective cohort study

**DOI:** 10.3389/fmed.2026.1733339

**Published:** 2026-02-27

**Authors:** Nicole Anna Dietzel, Maria Schmidt, Johannes Wiegand, Thomas Berg, Ronald Biemann, Ronny Baber, Michael Kluge, Kerstin Wirkner, Dirk Alexander Wittekind

**Affiliations:** 1 Institute of Laboratory Medicine, Clinical Chemistry and Molecular Diagnostics, University of Leipzig, Leipzig, Germany; 2 Medical Informatics Center—Division for Clinical AI and Translational Medicine, University of Leipzig Medical Center (MIC, MedKIT, ULMC), Leipzig, Germany; 3 Department of Medicine II, Division of Hepatology, University of Leipzig Medical Center, Leipzig, Germany; 4 Leipzig Medical Biobank, University of Leipzig, Leipzig, Germany; 5 Department of Psychiatry, Psychotherapy and Psychosomatics, Rudolf-Virchow-Klinikum Glauchau, Glauchau, Germany; 6 Department of Psychiatry and Psychotherapy, University of Leipzig, Leipzig, Germany; 7 Leipzig Research Centre for Civilization Diseases (LIFE), Leipzig University, Leipzig, Germany

**Keywords:** alcohol, growth differentiation factor 15, hepatic fibrosis, hepatic steatosis, insulin resistance, liver diseases, obesity

## Abstract

**Objective:**

Liver diseases represent a major global health burden. Growth Differentiation Factor 15 (GDF-15), a stress-induced cytokine, has been suggested to protect against fibrosis progression through neuro-metabolic-immunologic pathways and to regulate energy and lipid homeostasis, potentially influencing hepatic steatosis. This study evaluated the role of GDF-15 in steatosis and fibrosis, considering prior liver injury, alcohol intake, insulin resistance, and obesity.

**Design and methods:**

In this retrospective cohort study, 626 participants from a large population-based cohort were analyzed. Associations of baseline GDF-15, alcohol intake, FIB-4 score, and metabolic risk factors with hepatic steatosis and fibrosis over 6 years were examined using linear regression models.

**Results:**

In participants with elevated baseline FIB-4, the interaction of GDF-15 and FIB-4 was positively associated with follow-up liver stiffness (β = 0.47, *p* = 0.045). Interactions between GDF-15 and higher alcohol intake (3rd/4th quantiles) were negatively associated with stiffness (β = −1.68, p = 0.002; β = −1.43, *p* = 0.038). GDF-15 was positively associated with follow-up steatosis (β = 37.14, *p* = 0.006). Higher HOMA-IR (3rd/4th quantile) was linked to increased steatosis (β = 31.15, *p* = 0.032; β = 38.15, p = 0.023), whereas interactions of HOMA-IR × GDF-15 were inversely associated (β = −38.98, *p* = 0.008; β = −38.54, *p* = 0.019), suggesting a protective modulation.

**Conclusions:**

GDF-15 appears to modulate hepatic steatosis and fibrosis in individuals with metabolic or lifestyle risk factors, supporting its potential as a therapeutic target and warranting further investigation of the neuro-metabolic-immunologic axis.

## 1 Introduction

Chronic liver diseases are mostly driven by metabolic disorders such as obesity and insulin resistance (1) as well as by alcohol consumption (2). The pathogenesis of metabolic dysfunction associated steatotic liver disease (MASLD) is known to be due to increased *de novo* hepatic lipogenesis, which is caused as a result of heightening sugar consumption, leading to insulin resistance and impairment in glucose uptake (3). Over time, steatotic liver diseases and chronic exposure to toxic agents can promote liver fibrosis, a pathological process characterized by excessive collagen deposition and activation of hepatic stellate cells (HSCs) (4).

GDF-15 is implicated in a variety of metabolic- and alcohol related liver diseases (5–8) and signals via the GDNF receptor alpha-like (GFRAL), with RET acting as an essential co-receptor (9). This receptor complex is expressed exclusively in the area postrema and the nucleus of the solitary tract of the brainstem (10) where its activation induces an aversive visceral malaise state (11). Downstream of GFRAL/RET activation, GDF-15 elicits intracellular signaling cascades that potentially involve the AKT, ERK, and PLC-γ pathways, ultimately leading to reduced food intake, weight loss, reduced lipid accumulation, and improved glucose homeostasis. Accordingly, GDF-15 is proposed to act as a homeostatic regulator of energy intake, and preclinical evidence suggests that its specific binding to hindbrain-restricted GFRAL may enable relatively selective modulation of energy balance, supporting therapeutic exploration of the GDF-15/GFRAL axis in obesity (12).

As a biomarker of cellular stress (13), GDF-15 levels increase in response to inflammation and tissue damage (14) and display both pro- (15) and anti-inflammatory properties (16). Elevated GDF-15 levels have been linked to greater liver stiffness and higher risk of advanced fibrosis—independent of other metabolic risk factors (17).

Gut dysbiosis and impaired intestinal barrier function, triggered, or exacerbated by alcohol consumption and chronic metabolic diseases such as diabetes and obesity, promote chronic liver disease through dysregulation of the gut–liver axis. This dysfunction increases the translocation of gut-derived endotoxins, particularly lipopolysaccharides (LPS), to the liver, where they activate hepatic macrophages (Kupffer cells) and drive liver injury by inducing pro-inflammatory cytokines such as TNF-α, IL-6, and IL-1β (18–20).

Growth differentiation factor-15 (GDF-15) has emerged as a negative regulator of this inflammatory pathway by suppressing LPS-induced Kupffer cell activation and limiting inducible nitric oxide synthase (iNOS) expression and pro-inflammatory cytokine production, thereby exerting a protective immunomodulatory role in liver inflammation and ameliorating liver fibrosis (21, 22).

In the context of alcohol-related liver disease, mechanistic insights from animal models further support a protective role of GDF-15 in alcohol-induced liver injury and fibrosis via a neuro–metabolic–immunologic axis: Chronic alcohol consumption increases the hepatic influx of gut-derived catecholamines through the portal vein. These catecholamines activate β2-adrenergic receptors (ADRB2) in perivenous hepatocytes, promoting mitochondrial oxidative stress and inducing GDF-15 expression. GDF-15 subsequently upregulates ADRB2 expression in neighboring inflammatory Kupffer cells, leading to catecholamine-dependent ADRB2/PKA signaling and apoptosis of these immune cells, thereby limiting alcohol-associated hepatic inflammation (7).

Consistent with this regulatory function, clinical studies examining the role of GDF-15 in alcohol-related liver pathology have shown that chronic alcohol consumption increases GDF-15 levels in subjects with cardiovascular diseases or diabetes (23). Even acute binge drinking in healthy individuals elevates GDF-15 levels, which is associated with increased liver stiffness and the development of ultrasound-detectable hepatic steatosis (24), highlighting a potential role of GDF-15 as a stress-responsive factor in alcohol-related liver disease.

Metabolic dysfunction represents a key driver of chronic liver disease, with insulin resistance serving as a central pathophysiological link. By enhancing adipose tissue lipolysis and increasing circulating free fatty acids, insulin resistance promotes ectopic lipid accumulation in the liver, and resulting in hepatic steatosis (25, 26). Clinical studies have demonstrated a strong association between hepatic steatosis and insulin resistance (27). Importantly, the risk of developing clinically significant liver fibrosis increases with greater degrees of steatosis primarily in individuals with insulin resistance, whereas insulin-sensitive individuals display a consistently low fibrosis risk even in the presence of severe hepatic steatosis (28).

GDF-15 deficiency accelerates fibrosis and exacerbates obesity, while recombinant GDF-15 treatment mitigates liver damage (8, 21, 29). Mechanistically, GDF-15 contributes to the reduction of hepatic fat accumulation, and acts as a liver-secreted metabokine in high-fat fed mice (30), thus maintaining energy homeostasis by regulating carbohydrate metabolism (31) and improving insulin and glucose sensitivity (32). Animal studies indicate that GDF-15 attenuates hepatic steatosis by reducing oxidative stress–driven mitochondrial dysfunction (33) and modulates hepatic innate immune responses by limiting oxidative stress–induced inflammasome activation (34), thereby linking lipid overload to inflammation in the progression of hepatic steatosis and fibrosis.

Based on these results, the present study seeks to evaluate whether the protective effects of GDF-15 on liver fibrosis and steatosis can be observed in a large human cohort presenting with impaired liver function and/or metabolic risk factors.

We hypothesize that elevated levels of GDF-15 could give protection by attenuating the progress of liver fibrosis and steatosis, as assessed by transient elastography (FibroScan^®^) (35), particularly in subjects with pre-existing risk factors for liver steatosis, liver fibrosis or in the presence of metabolic or toxic stress.

## 2 Materials and methods

This study followed a retrospective cohort design, using data from the LIFE-Adult Study, a large prospective population-based study in Leipzig, Germany (36). At baseline (2011–2014), 10,000 adults aged 18–79 years were examined; 5,512 completed questionnaires and 1,799 underwent physical exams at follow-up (2017–2021) (36, 37). The study was approved by the Ethics Committee of the University of Leipzig, Germany, and has been performed in accordance with the ethical standards as laid down in the 1964 Helsinki Declaration and its later amendments or comparable ethical standards. Written informed consent was obtained from all participants.

Baseline data included demographics, lifestyle, BMI, alcohol, smoking, HbA1c, GDF-15, liver enzymes, albumin, bilirubin, and HOMA-IR. sociodemographic and medical data, including medications were necessary for inclusion. Liver elastography results from the follow-up were also necessary for inclusion. Perhaps it is worth clarifying that when the population-based study was initially designed in 2010, liver elastography was not included in the baseline assessment. It was introduced later during follow-up, which now allows us to correlate the presence of advanced fibrosis and steatosis observed during follow-up with risk factors assessed at baseline. Participants with recent or untreated hepatitis or systemic glucocorticoid/methotrexate use were excluded (38, 39).

Analyses targeted subgroups with: (1) elevated FIB-4, (2) or obesity (BMI >30 kg/m^2^) to examine the effect of baseline GDF-15 on liver stiffness and hepatic steatosis at follow-up. Supplement 1 provides further details on the selection process.

### 2.1 Assessment of baseline variables

GDF-15 serum samples were analyzed from samples stored in the vapor phase of liquid nitrogen at temperature below −150°C in sealed straws (Cryo Bio Systems IMV, L'Aigle, France) and Askion HS200 S storage devices (Gera, Germany). GDF-15 levels were quantified using an electrochemiluminescence immunoassay (ECLIA) on a Cobas 8000 automated laboratory analyser (Roche Diagnostics, Mannheim, Germany) in accordance with the manufacturer's instructions (Roche Diagnostics, Mannheim, Germany). According to the manufacturer, the assay has a measuring range of 400–20,000 pg/mL, with a lower limit of quantification of 400 pg/mL.

The FIB-4 score was calculated using the formula by Sterling et al. (40). Age-specific thresholds were applied to define fibrosis risk: >1.3 for individuals <65 years and >2.0 for those ≥65 years (41).

Baseline HOMA-IR was calculated and stratified using the formula and cut-off-values proposed by Matthews et al. (42). BMI at baseline was calculated as weight in kilograms divided by the square of height in meters, applying established cut-offs for obesity (43). For further analyses samples were stratified in HOMA-IR quantiles.

Alcohol intake over the past 12 months was assessed using a validated, self-administered Food Frequency and Alcohol Questionnaire (FFQ). Participants reported consumption frequency and usual quantity of drinks, from which average daily alcohol intake (g/day) at baseline was calculated. For analysis, participants were stratified by alcohol intake quantiles.

### 2.2 Measurement of follow-up variables

At follow-up, liver stiffness (LSM, kPa) and steatosis (CAP, dB/m) were assessed by FibroScan^®^ in fasting state and ten valid measurements were recorded for each participant, in accordance with current recommendations (44).

### 2.3 Confounders

Models were adjusted for age, sex, BMI, HbA1c, and smoking, all known to be associated with GDF-15 (45). Smoking was included only in the steatosis models, as cigarette smoking has been associated primarily with an increased risk of NAFLD, but not with increased liver stiffness in population-based studies (46).

### 2.4 Statistical analysis

Skewed variables were log-transformed. In the FIB-4 subgroup, linear regression tested associations of alcohol intake quantiles, FIB-4 and baseline GDF-15, and interaction effects of GDF-15 with FIB-4 or alcohol quantiles on follow-up liver stiffness, adjusting for age, sex, BMI, HbA1c, and GGT. For steatosis, models assessed baseline GDF-15 interactions with HOMA-IR in the obese subgroup, adjusting for age, sex, HbA1c, and smoking. Analyses were conducted in R version 4.4.2 (R Project for Statistical Computing, https://scicrunch.org/resolver/RRID:SCR_001905).

## 3 Results

### 3.1 Descriptive statistics

The study included 626 subjects categorized at baseline according to FIB-4-score and obesity status. The average time between baseline and follow-up measurement was 6.40 years (SD = 0.40; range: 4.88–8.67). In the subgroup with elevated FIB-4 (*n* = 220; mean age 65.4 ± 8.6 years), the mean baseline GDF-15 concentration was 936.87 pg/mL (SD = 581.56). Average alcohol intake was 15.96 g/day (SD = 20.35). The mean baseline FIB-4 score was 2.15 (SD = 0.71). At follow-up, the mean liver stiffness measurement was 5.72 kPa (SD = 3.29). Additional descriptive information is provided in Table 1 and Supplementary Table 1.

**Table 1:** Descriptive data elevated FIB-4 sample.

			**Baseline**					**Follow-up**				
	**Variable**	**n**	**mean**	**sd**	**median**	**min**	**max**	**mean**	**sd**	**median**	**min**	**max**
Subjects with FIB-4 >1.3 (<65 years) or FIB >2.0 (≥ 65 years)	FIB-4	220	2.15	0.71	2.10	1.31	7.23	2.02	0.73	1.94	0.38	5.97
	GDF15 [pg/ml]	220	936.87	581.56	785.50	400.00	6185.00	NA	NA	NA	NA	NA
	Alcohol [g/day]	220	15.96	20.35	8.47	0.03	123.20	NA	NA	NA	NA	NA
	Age [years]	220	65.44	8.62	64.86	28.89	79.57	71.80	8.62	71.25	35.37	85.75
	BMI	220	27.16	3.82	26.67	18.72	39.07	27.03	3.72	26.58	18.15	41.86
	HbA1c [%]	220	5.46	0.54	5.38	4.55	9.15	NA	NA	NA	NA	NA
	GGT [mikrokat/l]	220	0.61	0.61	0.42	0.16	6.60	0.53	0.56	0.34	0.12	4.49
	liverstiffness [kPa]	220	NA	NA	NA	NA	NA	5.72	3.29	5.00	2.20	28.00
		controlled attenuation parameter (CAP) [dB/m]	220	NA	NA	NA	NA	NA	274.80	55.74	272.50	100.00	400.00

In the subgroup with elevated BMI (*n* = 113; mean age 63.15 ± 10.83 years), baseline GDF-15 was 924.00 pg/mL (SD = 461.22), and HOMA-IR was 4.49 (SD = 6.38). At follow-up, the mean CAP value was 299.72 dB/m (SD = 55.34). Additional information is provided in Table 2 and Supplementary Table 1. Quantiles of alcohol intake and HOMA-IR were constructed for statistical analyses (Supplementary Table 2).

**Table 2:** Descriptive data BMI > 30 sample at baseline.

			**Baseline**					**Follow-up**				
	**Variable**	**n**	**mean**	**sd**	**median**	**min**	**max**	**mean**	**sd**	**median**	**min**	**max**
Subjects with BMI ≥ 30	HOMA-Score	113	4.49	6.38	3.38	0.42	65.77	NA	NA	NA	NA	NA
	GDF15 [pg/ml]	113	924.65	461.22	845.00	400.00	2 810.00	NA	NA	NA	NA	NA
	age [years]	113	63.15	10.83	66.11	23.50	77.57	69.54	10.76	72.58	30.31	83.69
	BMI	113	32.42	2.03	31.70	30.01	39.07	31.74	2.98	31.65	23.47	41.86
	HbA1c [%]	113	5.53	0.51	5.44	4.67	7.36	NA	NA	NA	NA	NA
	GGT [mikrokat/l]	113	0.62	0.46	0.49	0.16	3.37	0.55	0.43	0.45	0.12	2.65
	liverstiffness [kPa]	113	NA	NA	NA	NA	NA	6.17	3.55	5.40	2.20	28.00
		controlled attenuation parameter (CAP) [dB/m]	113	NA	NA	NA	NA	NA	299.72	55.34	297.00	100.00	400.00

### 3.2 Main findings

**Figure 1:**
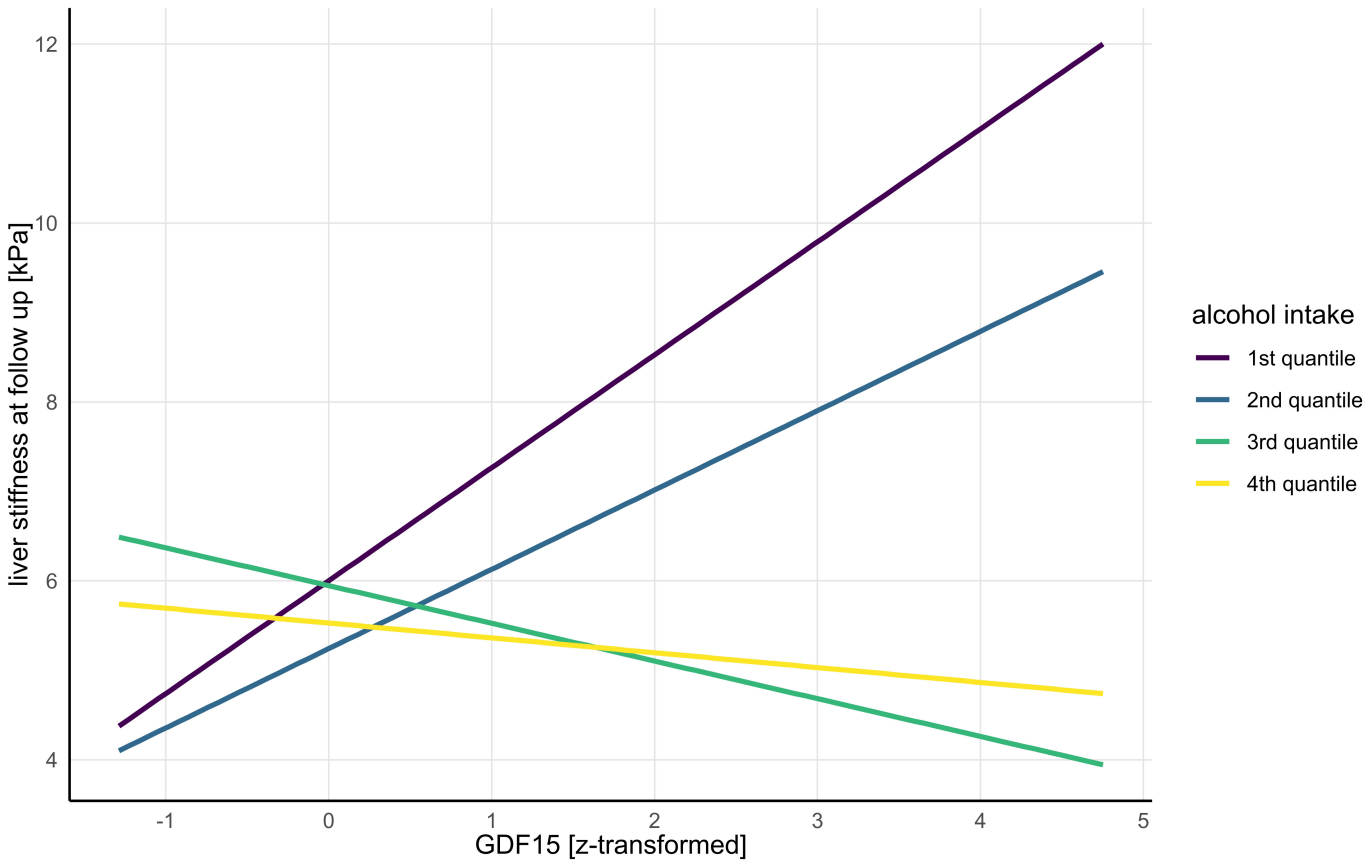
Interaction of alcohol intake quantiles and baseline GDF-15 on liver stiffness at follow-up. *GDF-15, growth differentiation factor 15*.

Among subjects with elevated FIB-4-scores at baseline, baseline GGT was significantly associated with liver stiffness measurement at follow-up (ß = 0.49, 95% CI: 0.060–0.916, SE = 0.22, *t* = 2.25, *p* = 0.026). Baseline FIB-4-score and baseline GDF-15 did not show significant main effects on liver stiffness measurement at follow (*p* > 0.05). However, their interaction showed a significant positive association on liver stiffness measurement at follow up (ß = 0.47, 95% CI: 0.0109–0.933, SE = 0.23, *t* = 2.02, *p* = 0.045). The interaction between baseline GDF-15 and alcohol intake quantiles revealed significant negative associations with liver stiffness measurement at follow-up for the third (ß = −1.68, SE = 0.55, *t* = −3.08, *p* = 0.002) and forth quantile (ß = −1.43, 95% CI: −2.78 to −0.082, SE = 0.68, *t* = −2.09, *p* = 0.038) of alcohol intake (Table 3, Figure 1). Subjects in the third quantile reported daily alcohol intake between 8.53 g/day and 19.92 g/day with a mean alcohol intake of 14.08 g/day (SD = 3.15) while subjects in the fourth quantile consumed between 21.21 g/day and 123.20 g/day alcohol, with an average intake of 43.57 g/day (SD= 23.21).

**Table 3:** Interaction Model of GDF15^*^FIB-4 and GDF-15^*^alcohol quantile on liver stiffness measurement at follow-up.

**Variable**	**Estimate**	**Std. error**	***t* value**	**Pr(>|t|)**	**Lower 95% CI**	**Upper 95% CI**
(Intercept)	5.88	0.61	9.69	<0.001^***^	4.68	7.08
z.GDF15	0.84	0.50	1.67	0.096	−0.151	1.84
z.FIB-4	0.10	0.24	0.43	0.669	−0.376	0.585
Alkohol_Quantil2	−0.76	0.63	−1.21	0.228	−2.00	0.480
Alkohol_Quantil3	−0.05	0.66	−0.08	0.934	−1.36	1.25
Alkohol_Quantil4	−0.47	0.70	−0.67	0.503	−1.86	0.916
z.GGT_T1	0.49	0.22	2.25	0.026^*^	0.060	0.916
z.Alter	−0.42	0.42	−0.99	0.325	−1.25	0.415
Sex (Female)	−0.65	0.53	−1.24	0.218	−1.69	0.389
z.BMI	0.21	0.21	0.96	0.337	−0.215	0.625
z.HBA1C	0.42	0.22	1.92	0.056	−0.0109	−0.856
z.FIB4 ^*^ z.GDF15	0.47	0.23	2.02	0.045^*^	0.0109	0.933
z.GDF15^*^ Alkohol_Quantil2	−0.38	0.61	−0.61	0.540	−1.58	0.832
z.GDF15^*^ Alkohol_Quantil3	−1.68	0.55	−3.08	0.002^**^	−2.76	−0.607
z.GDF15^*^ Alkohol_Quantil4	−1.43	0.68	−2.09	0.038^*^	−2.78	−0.082

^*^Model FIB-4 - FIB-4 > 1.3 (<65 years) or FIB-4 >2.0 (≥ 65 years): Interaction of GDF15 x FIB-4 and GDF15 x alcohol quantile on liver stiffness: *N* = 220; *R*^2^ = 0.191; adjusted *R*^2^ = 0.136; *F* (8, 211) = 7.80, *p* < 0.001. ^*^p < 0.05; ^**^p < 0.01; ^***^p < 0.001.

A weak significant positive correlation emerged between baseline GDF-15 and liver stiffness at follow-up (Spearman's ρ = 0.262, *p* < 0.001). Predictors of baseline GDF-15 in the subgroup with elevated FIB-4 score included baseline FIB-4 score (ß = 105.00, 95% CI: 23.2–187.0, SE = 41.47, *t* = 2.53, *p* = 0.012), age (ß = 234.73, 95% CI:98.5–371.0, SE = 69.11, *t* = 3.40, *p* = 0.001), active smoking (ß = 580.95, 95% CI: 264.0–898.0, SE = 160.81, *t* = 3.61, *p* < 0.001) and HbA1c (ß = 85.90, 95% CI: 16.1–156.0, SE = 35.41, *t* = 2.43, *p* = 0.016).

**Figure 2:**
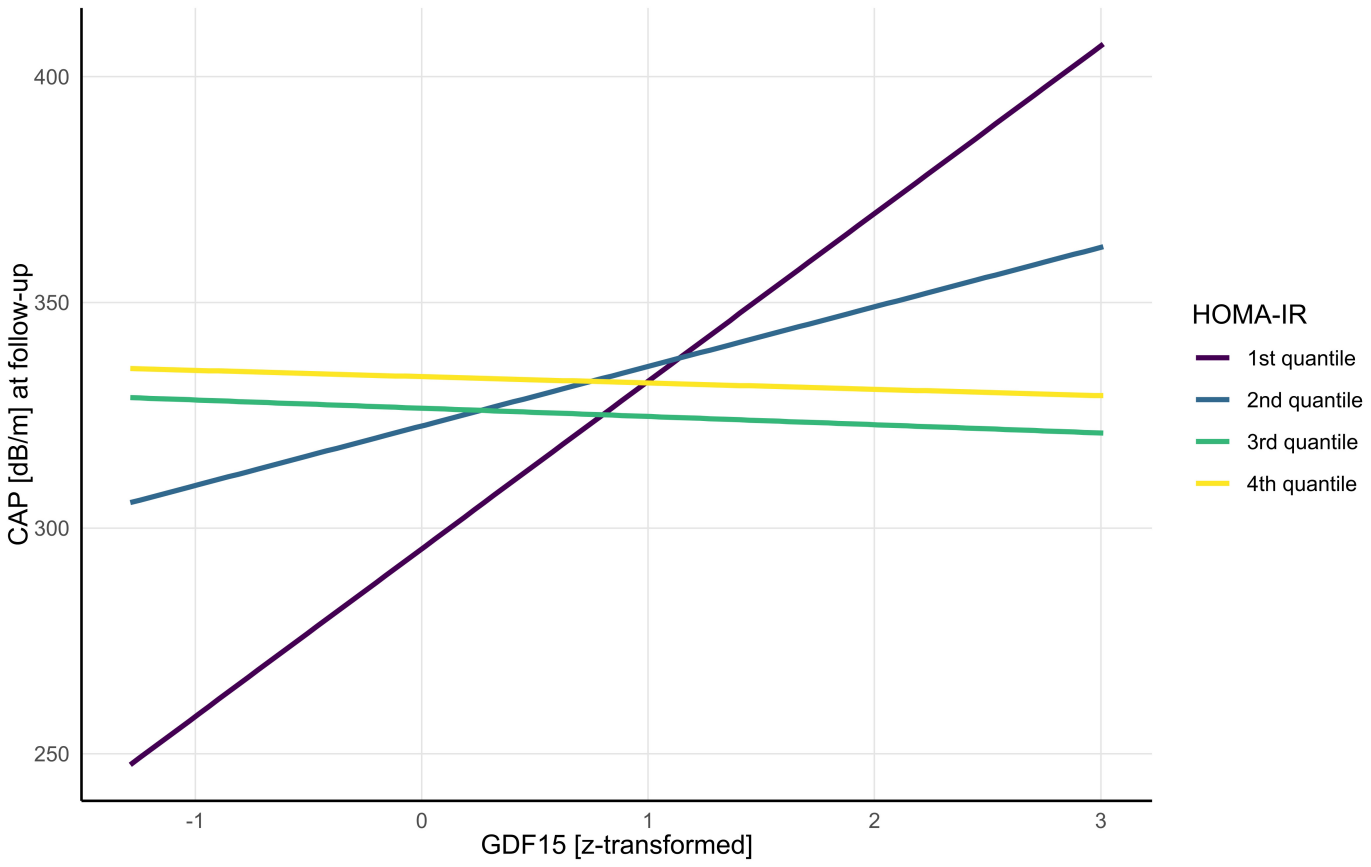
Interaction of HOMA-IR quantiles and baseline GDF-15 on hepatic steatosis (CAP) at follow-up. *CAP, controlled attenuation parameter; HOMA-IR, homeostatic model assessment of insulin resistance; GDF-15, growth differentiation factor*.

In the obese subgroup, baseline GDF-15 (ß = 37.14, 95% CI: 11.0–63.2, SE = 13.15, *t* = 2.82, *p* = 0.006) as well as the third (ß = 31.15, 95% CI: 2.71–59.6, SE = 14.33, *t* = 2.17, *p* = 0.032) and fourth quantile of HOMA-IR (ß = 38.15, 95% CI: 5.46–70.8, SE = 16.48, *t* = 2.32, *p* = 0.023) were positively associated with CAP at follow-up. The interaction between GDF-15 and baseline HOMA-IR on CAP varied by HOMA-IR quantile. Significant negative interactions were observed in the third (ß = −38.98, 95% CI: −67.3 to −10.6, SE = 14.29, *t* = −2.73, *p* = 0.008) and fourth quantile of HOMA-IR [ß = −38.54, 95% CI: −70.5 to −6.58, SE = 16.11, *t* = −2.39, *p* = 0.019 (Table 4, Figure 2)].

**Table 4:** Interaction model of GDF15 and HOMA-IR quantile on CAP at follow-up.

**Variable**	**Estimate**	**Std. error**	***t* value**	**Pr(>|t|)**	**Lower 95% CI**	**Upper 95% CI**
(Intercept)	297.28	12.38	24.02	<0.001	273.0	322.0
z.GDF15	37.14	13.15	2.82	0.006^**^	11.0	63.2
HOMA-IR_Quantil2	27.23	14.08	1.93	0.056	−0.702	55.2
HOMA-IR_Quantil3	31.15	14.33	2.17	0.032^*^	2.71	59.6
HOMA-IR_Quantil4	38.15	16.48	2.32	0.023^*^	5.46	70.8
z.Alter	−20.06	9.56	−2.10	0.038^*^	−39.0	−1.10
Sex (Female)	−28.19	10.57	−2.67	0.009^**^	−49.2	−7.22
Smoking factor: former smoker	−6.05	10.82	−0.56	0.577	−27.5	15.4
Smoking factor: active smoker	−41.25	20.19	−2.04	0.044^*^	−81.3	−1.21
z.HBA1C	9.63	5.43	1.77	0.079	−1.15	20.4
z.GDF15^*^ HOMA-IR_Quantil2	−23.95	17.16	−1.40	0.166	−58.0	10.1
z.GDF15^*^ HOMA-IR_Quantil3	−38.97	14.29	−2.73	0.008^**^	−67.3	−10.6
z.GDF15^*^ HOMA-IR_Quantil4	−38.54	16.11	−2.39	0.019^*^	−70.5	−6.58

Model BMI ≥ 30: Interaction of GDF15 x HOMA-IR quantile on CAP at follow up: *N* = 113; *R*^2^ = 0.265; adjusted *R*^2^ = 0.177; F (12, 100) = 3.011, *p* = 0.001). ^*^p < 0.05; ^**^p < 0.01.

HOMA-IR values in the third quantile ranged from 3.38 to 4.93 (mean: 4.11; SD = 0.50), while those in the fourth quantile ranged from 5.04 to 65.77 (mean: 9.45; SD = 11.46).

Additionally, sex, smoking status, and age showed significant associations with CAP at follow-up. Female sex (β = −28.19, 95% CI: −49.2 to −7.22, SE = 10.57, *t* = −2.67, *p* = 0.009), active smoking (vs. never smoking; β = −41.25, 95% CI: −81.3 to −1.21, SE = 20.18, *t* = −2.04, *p* = 0.044) and age (β = −20.06, 95% CI: −39.0 to −1.10, SE = 9.55, *t* = −2.10, *p* = 0.038) were associated with lower CAP values.

## 4 Discussion

In this study, we investigated the moderating effects of baseline GDF-15 on liver fibrosis and steatosis at follow-up in subjects with pre-existing risk factors. It is important to emphasize that we are not simply reporting the well-established association between elevated GDF-15 and liver injury. Rather, our data suggests that high baseline levels of GDF-15 are linked to more favorable trajectories under specific risk conditions. Interestingly, among participants with high FIB-4 scores, GDF-15 appeared to attenuate fibrosis in those with higher alcohol intake, pointing toward a possible alcohol-consumption dependent protective effect. A similar pattern was evident for hepatic steatosis. While both GDF-15 and HOMA-IR were positively associated with steatosis in individuals with obesity, their interaction showed an inverse relationship. This finding indicates that GDF-15 may counterbalance the detrimental impact of insulin resistance, acting in a compensatory or protective manner. Taking together, these results highlight significant interaction effects of GDF-15 with alcohol consumption and insulin resistance on fibrosis and steatosis in already vulnerable groups. This suggests that GDF-15 may exert a protective influence specifically in the presence of metabolic or toxic risk factors, whereas in the absence of such conditions (e.g., no alcohol risk intake, no insulin resistance, and no obesity), we did not observe protective interactions.

The observed protective effect of elevated GDF-15 levels in individuals with high alcohol intake supports disease models derived from animal experiments, which suggest the existence of a neuro-metabolic-immune axis and immunomodulatory effects mediated by GDF-15 that inhibit hepatic fibrogenesis and inflammation (7, 21, 47). Mechanistically, GDF-15 limits hepatic inflammation and fibrosis by regulating tissue cellular immunity through modulation of regulatory T cells, enhanced anti-inflammatory IL-10 production and suppression of T-cell effector responses (48, 49). Furthermore, GDF-15 promotes the metabolic reprogramming of hepatic macrophages toward an oxidative phosphorylation-dependent anti-inflammatory phenotype (M2 polarization), thereby reducing inflammatory macrophage infiltration, pro-inflammatory (M1) polarization, and proinflammatory cytokine secretion, ultimately attenuating liver inflammation, and fibrogenesis (21, 50). In line with our results Kim et al. described a neuro-metabolic-immune axis in which the apoptosis of Kupffer cells leads to a reduction in proinflammatory cytokine production, and consequently, to decreased hepatic inflammation in the context of alcohol-associated liver disease: Alcohol-fed mice showed increased GDF-15 levels in perivenous hepatocytes, along with elevated blood catecholamine concentrations originating from the intestine. Within the liver, catecholamines exert their effects through the activation of adrenergic receptors, particularly the β2-adrenergic receptor (ADRB2) on hepatocytes and Kupffer cells. ADRB2 activation in hepatocytes triggers the expression of GDF-15, which then activates mitochondrial CYP2E1 and results in an increase in oxidative stress. In response to oxidative stress, hepatocyte-derived GDF-15 plays a dual role: it increases the expression of ADRB2 on Kupffer cells while, at the same time, inducing their apoptosis. In a small group of patients (*n* = 18) with early-stage ALD, the patients who had high levels of catecholamines in their stool and blood and increased perivenous expression of GDF-15 showed an enhanced frequency of apoptotic Kupffer cells (7). Further evidence supports this protective role of GDF-15 against alcohol-related liver damage. Mice lacking GDF-15 developed more severe liver damage and fibrosis after six weeks of alcohol exposure, compared to wild-type controls (8). These findings suggest that GDF-15 not only serves as a biomarker reflecting cellular stress but also plays an active role in regulating hepatic inflammation by modulating T-cell signaling, macrophage polarization and preventing the survival of proinflammatory Kupffer cells, thereby mitigating the progression of hepatic inflammation and fibrosis.

Researchers have reported similar injury-modulating and antifibrotic effects of GDF-15 in extrahepatic tissues. In a mouse model of LPS-induced sepsis, GDF-15 deficiency led to increased neutrophil infiltration and inflammation in cardiac and renal tissue, while GDF-15 transgenic mice showed reduced apoptosis and tissue necrosis (51). In models of chronic kidney injury using unilateral ureter obstruction, GDF-15 suppressed the proliferation and survival of activated renal fibroblasts by downregulating the oncogene N-Myc, leading to cell cycle arrest and apoptosis (52). Pulmonary fibrosis models provide additional support: recombinant GDF-15 reduced fibroblast activity *in vitro* and improved lung architecture and collagen deposition *in vivo*. Lower GDF-15 levels in patients with interstitial lung diseases further suggest a protective role in fibrosis progression (53).

Interestingly, studies in cancer immunotherapy show that blocking GDF-15 with Visugromab restored anti-PD-1/PD-L1 sensitivity in patients with solid tumors, including HCC, likely by lifting suppression of inflammatory signaling in liver macrophages (54). Together, these findings point to a dual role of GDF-15: while its suppression of inflammation may hinder antitumor immunity, it appears beneficial in chronic liver disease by protecting against fibrosis. This effect is also seen in kidney and lung models. GDF-15 may thus function in a dual role: first, as a signaling agent to the central nervous system (CNS) to induce a state of malaise in inflammation and secondly, as a local regulator of inflammation and fibrosis across multiple organs.

However, when interpreting GDF-15′s antifibrotic role in the context of alcohol intake, it is important to note the dose-response relationship. The interaction between GDF-15 levels and alcohol consumption was evident only in the third and fourth quantile of alcohol intake, which corresponds to a daily alcohol intake of 8.53–19.92 g/day in the third quantile and 21.21–123.20 g/day in the fourth quantile. Notably, alcohol intake in the forth quantile exceeds the upper limits recommended by European guidelines, which advise a maximum of 20 g/day for men and 10 g/day for women (55). Current World Health Organization statement emphasizes the harmful effects of alcohol from the very first consumption and recommends that complete abstinence from alcohol is the only approach beneficial to health (56). In our study, we did not observe a direct association between GDF-15 levels and alcohol consumption, suggesting that GDF-15 is not automatically upregulated in response to alcohol intake or alcohol-related pathologies. One study among subjects aged 65 years and older found no significant association between average lifetime alcohol consumption and GDF-15 levels in participants without cardiovascular disease or diabetes mellitus. However, in high-risk drinkers (defined as >40 g of alcohol/day for men and >20 g of alcohol/day for women), GDF-15 levels showed an elevation of 0.27% for every additional gram of alcohol consumed per day (23). These findings suggest that alcohol must cause a certain threshold of cellular damage before it can activate the neuro-metabolic-immune axis that drives GDF-15 expression. This pattern is in line with how GDF-15 levels respond to pathological states: GDF-15, as a stress-responsive cytokine, is secreted in small amounts under physiological conditions, with an average level of 450 pg/ml (57), but can rise dramatically to 10,000–100,000 pg/ml in pathological states in humans (58, 59). In this respect, it has a similar dynamic as, for example, CRP or IL-6. Several toxic and mechanical stimuli have been shown to influence GDF-15 levels. Partial hepatectomy, as well as intraperitoneal injection of carbon tetrachloride (CCl4) or ethanol resulted in elevated GDF-15 mRNA levels within 30 min to 12 h after treatment in murine liver models (60). Furthermore, palmitate treatment increased GDF-15 mRNA expression in Kupffer cells (17). High levels of GDF-15 have been detected in both cirrhotic human liver tissues and in mice exposed to carbon tetrachloride and thioacetamide to experimentally induce liver fibrosis, particularly in cirrhotic tissue and activated hepatic stellate cells (15). Research on alcohol-related liver disease showed that chronic alcohol use raised GDF-15 levels in the elderly, particularly in subjects with cardiovascular disease or diabetes mellitus, whereas moderate alcohol consumption did not significantly relate to GDF-15 levels in healthy subjects (23). Nevertheless, it remains unclear whether elevated alcohol consumption *per se* is sufficient to stimulate GDF-15 production and its possible protective effects—or whether, as our results suggest, only when cellular damage is induced by alcohol consumption.

In contrast to the beneficial interaction effects observed in our study, GDF-15 showed a reinforcing effect on liver fibrosis in individuals who had elevated FIB-4 scores but no additional risk factors. This finding aligns with previous research demonstrating that GDF-15 levels correlate with liver stiffness (Spearman's ρ = 0.525, *p* < 0.001). Several studies reported that GDF-15 is significantly higher in individuals with advanced fibrosis and advanced severity of chronic liver disease (61) and that GDF-15 correlates with the severity of hepatic lobular inflammation (17). As a result, researches have discussed GDF-15 as a biomarker for predicting liver cancer occurrence in patients with elevated FIB-4 in metabolic associated liver disease (62, 63). Other findings confirmed that individuals in the highest quartile of GDF-15 levels faced an increased risk of advanced fibrosis, irrespective of other metabolic risk factors (17). Contrary, GDF-15 expression is decreased in livers from patients and mice with fibrosis or cirrhosis (21), which may reflect a ceiling effect beyond which the immunomodulatory mechanisms of GDF-15 are no longer able to act compensatory to limit hepatic inflammation and fibrosis. Importantly, adding GDF-15 to existing non-invasive fibrosis markers—such as the FIB-4 score—results in an improvement of diagnostic accuracy for multiple hepatic outcomes—including fibrosis, cirrhosis, and liver-related mortality (64). These findings indicate that GDF-15 may amplify fibrosis risk in individuals already identified as at-risk through elevated FIB-4 scores. Thus, a constellation of structural risk (FIB-4) and GDF-5′s stress signaling at the cellular level could identify patients at highest risk of fibrotic liver disease. These findings together support a potential amplifying role of GDF-15 in fibrogenesis.

Our study suggests not only a protective effect against liver fibrosis in subjects with high alcohol intake, but also a protective role against liver steatosis in obese individuals with advanced insulin resistance. Although HOMA-IR and GDF-15 were each positively associated with CAP in the obese subset, their interaction was negatively associated with CAP, indicating that GDF-15 may act as a metabolic stress–responsive modulator, attenuating hepatic lipid accumulation specifically under conditions of pronounced insulin resistance.

In the present study, we observed positive main effects of both HOMA-IR and GDF-15 on CAP, indicating that insulin resistance and metabolic stress–related signaling are key determinants of hepatic fat accumulation in obese individuals. These findings are consistent with prior evidence linking insulin resistance to the severity of steatosis in MASLD (27, 65) and demonstrating dynamic increases in GDF-15 in parallel with intrahepatic lipid accumulation (66).

Mechanistically, insulin resistance promotes hepatic triglyceride accumulation through increased adipose tissue lipolysis and enhanced free fatty acid flux to the liver, combined with hyperinsulinemia- and hyperglycemia-driven *de novo* lipogenesis via SREBP-1c (32). Concurrent inhibition of mitochondrial fatty acid oxidation through malonyl-CoA-mediated suppression of CPT-1 further favors hepatic lipid accumulation (25). As adiposity and accompanying insulin resistance promote increased hepatic triglyceride synthesis, lipotoxic triglyceride-derived metabolites induce endoplasmic reticulum stress and macrophage infiltration, collectively triggering robust GDF-15 expression (6), a finding supported by clinical studies demonstrating that hyperinsulinemia acutely increases GDF-15 expression (67).

Previous studies have demonstrated that CAP is more strongly associated with insulin resistance than with general adiposity, and that this relationship persists after adjustment for BMI (68). In line with these findings, our exploratory analyses revealed a significant positive association between baseline HOMA-IR and follow-up CAP (β = 12.71, 95% CI 3.05–22.40, *p* < 0.01), independent of age, sex, and BMI, supporting the concept that insulin resistance contributes to hepatic fat accumulation beyond the degree of overall adiposity (69). Although the observed correlation between HOMA-IR and CAP in our obese subset was weaker (*r* = 0.30, *p* < 0.05) than that reported in cross-sectional cohorts the direction of the association was consistent (68, 70). Moreover, higher HOMA-IR quantiles were associated with higher CAP values, as reported in the results section, a finding which is consistent with the established pathophysiological role of insulin resistance in the development of steatosis (25). Previous studies have examined both insulin resistance as a determinant of liver fat and liver fat as a determinant of insulin resistance (68, 70), suggesting a bidirectional relationship. However, due to our study design, we were only able to assess the association from baseline HOMA-IR to follow-up CAP.

Beyond the main effects, we identified a significant interaction between GDF-15 and HOMA-IR on CAP, which was restricted to individuals with advanced insulin resistance. Previous experimental studies have characterized GDF-15 as a protective liver-derived metabokine (33), with GDF-15 deficiency exacerbating steatosis under high-fat diet conditions (34). Mechanistically, GDF-15 has been shown to counteract hepatic lipid accumulation through enhanced catabolic signaling (71), reduced SREBP-1c expression (30), and β-adrenergic receptor–mediated pathways in adipose tissue and liver, independent of weight loss (72, 73). Chronic activation of β-adrenergic signaling may additionally improve peripheral glucose uptake and insulin sensitivity, thereby attenuating metabolic stress and hepatic lipid deposition (72). This β-adrenergic signaling, stimulated by adrenaline and noradrenaline, may resemble the protective mechanism of GDF-15 observed in liver fibrosis, as described by Kim et al. (7). Furthermore, immunomodulatory effects of GDF-15, including suppression of oxidative stress–induced mitochondrial dysfunction (74) and inhibition of AIM2 inflammasome activation, may contribute to reduced hepatic inflammation and steatosis progression (34). Importantly, the interaction between GDF-15 and HOMA-IR was observed only in HOMA-IR quantiles 3 and 4, corresponding to prediabetic and diabetic metabolic states, whereas no interaction was detected in mildly insulin-resistant individuals. This pattern suggests a threshold or dose–response effect, whereby a certain degree of metabolic stress is required to unmask the protective influence of GDF-15 on hepatic fat accumulation.

Notably, emerging evidence indicates that GDF-15 may act synergistically with FGF-21, another liver- and adipose tissue–derived metabolic regulator (30, 74). In mice, GDF-15 overexpression upregulates FGF-21 mRNA and serum levels, and exogenous GDF-15 administration increases FGF-21 levels in murine models (75), suggesting a coordinated mechanism by which these two metabokines enhance energy homeostasis, improve insulin sensitivity (74) and protect against hepatic steatosis and inflammation (76). High-fat diet–fed mice exhibited elevated blood glucose, insulin, and HOMA-IR, all of which were significantly reduced by combined GDF-15 and FGF-21 treatment, along with amelioration of obesity and hyperlipidemia. Hepatic protection was also evident: ALAT levels, which were elevated in GDF-15–treated chow-fed mice, were reduced by approximately 50% in high-fat diet–fed mice receiving both GDF-15 and FGF-21, and histological analyses confirmed markedly reduced hepatic steatosis (75). It is therefore possible that some of the effects observed in our study may be mediated, at least in part, by FGF-21 or by the interplay between GDF-15 and FGF-21, an aspect that warrants further investigation.

From a clinical perspective, our study suggests that GDF-15 is not a simple linear biomarker of liver disease severity but reflects a context-dependent stress response, particularly under metabolic or toxic burden. The absence of a uniform effect across all conditions, together with significant interactions with alcohol intake and insulin resistance, indicates that GDF-15 may influence the trajectory of liver disease progression rather than merely predicting outcomes. Higher GDF-15 levels were linked to less liver stiffness in individuals with higher alcohol intake and appeared to attenuate the impact of insulin resistance on hepatic fat accumulation in obese subjects, which may help explain heterogeneous hepatic changes among subjects with similar risk profiles. In those with elevated baseline fibrosis risk, increased GDF-15 likely represents an active compensatory response rather than inactive disease, supporting its potential utility in stratifying at-risk individuals. From a therapeutic standpoint, the highly specific binding of GDF-15 to its receptor GFRAL, which is expressed in a restricted manner within the hindbrain, renders this pathway pharmacologically attractive by enabling targeted modulation with potentially limited off-target effects (9, 12). Although GDF-15 analogs are currently being studied in preclinical and early-phase trials for obesity (77), further research is needed to clarify their relevance for liver disease treatment.

## 5 Strengths and limitations

This study is the first to examine the mediating role of elevated baseline GDF-15 secretion on hepatic steatosis and fibrosis at follow-up in a large human population with pre-existing fibrosis risk and metabolic or alcohol-related risk factors. The retrospective design necessarily limits causal inference. As liver elastography was not available at baseline, fibrosis risk was estimated using the FIB-4 score, whereas follow-up assessment relied on transient elastography; this methodological difference precludes direct comparability. Alcohol consumption was assessed using a self-administered food frequency and alcohol questionnaire; however, recall bias and underreporting—particularly of alcohol intake due to social desirability bias—cannot be excluded. Few participants reported severe alcohol intake, which may affect generalizability to alcohol-induced liver injury. The mean length of follow-up of 6.4 years, may introduce possible confounding from temporal changes in toxic and metabolic risk factors affecting GDF-15 levels, hepatic metabolism, and morphological changes.

## 6 Conclusion

In this study, we observed context-dependent associations between GDF-15 and liver steatosis and fibrosis in subjects with metabolic and toxic risk factors. In participants without additional lifestyle-related risks, higher GDF-15 levels were not associated with reduced liver injury and may have been associated with greater fibrosis and steatosis. In contrast, in subgroups with additional risk—such as increased insulin resistance in obesity or elevated alcohol intake in the high FIB-4 group—GDF-15 was inversely associated with disease progression, in line with findings from animal studies. However, given the observational nature of these findings, causal relationships cannot be inferred, and future prospective human studies are needed to clarify whether GDF-15 acts as a compensatory protective factor or contributes to maladaptive stress responses depending on the clinical context.

## Data Availability

The raw data supporting the conclusions of this article will be made available by the authors, without undue reservation.
