# Supplementary material for: Growth Differentiation Factor 15 (GDF-15) as a modulator of hepatic steatosis and fibrosis: insights from a 6-year retrospective cohort study
Source: Front Med (Lausanne). 2026 Feb 27;13:1733339. doi: 10.3389/fmed.2026.1733339 (PMC12982360; doi:10.3389/fmed.2026.1733339)
Supplement: Supplementary file 1 [file Data_Sheet_1.docx]

Supplementary Material

#
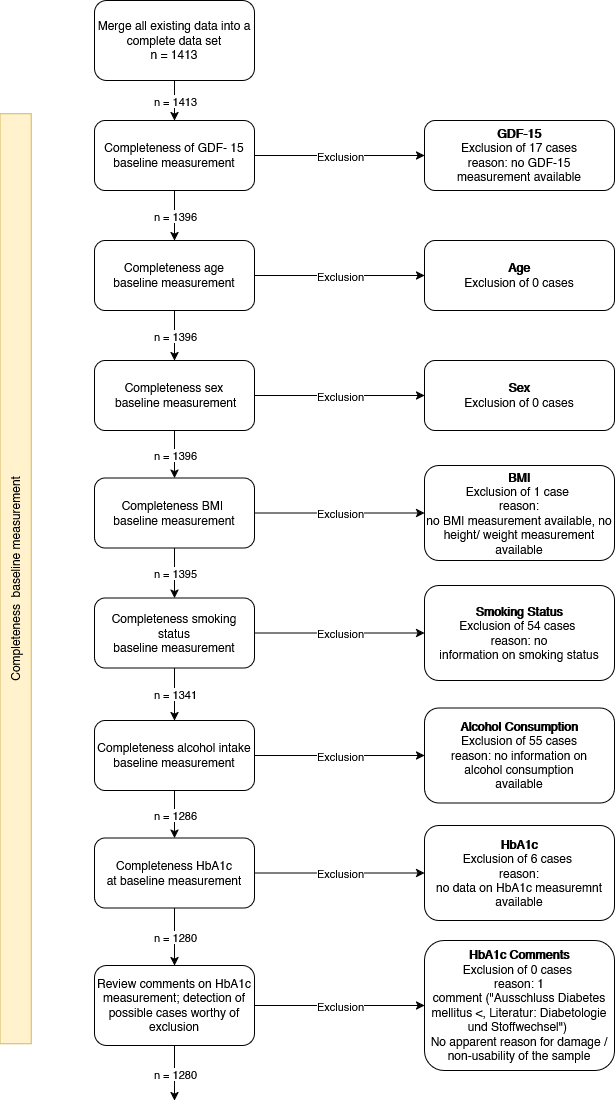
Supplement 1 – Data selection procedure

**Supplementary Figure 1** Data Selection I

**
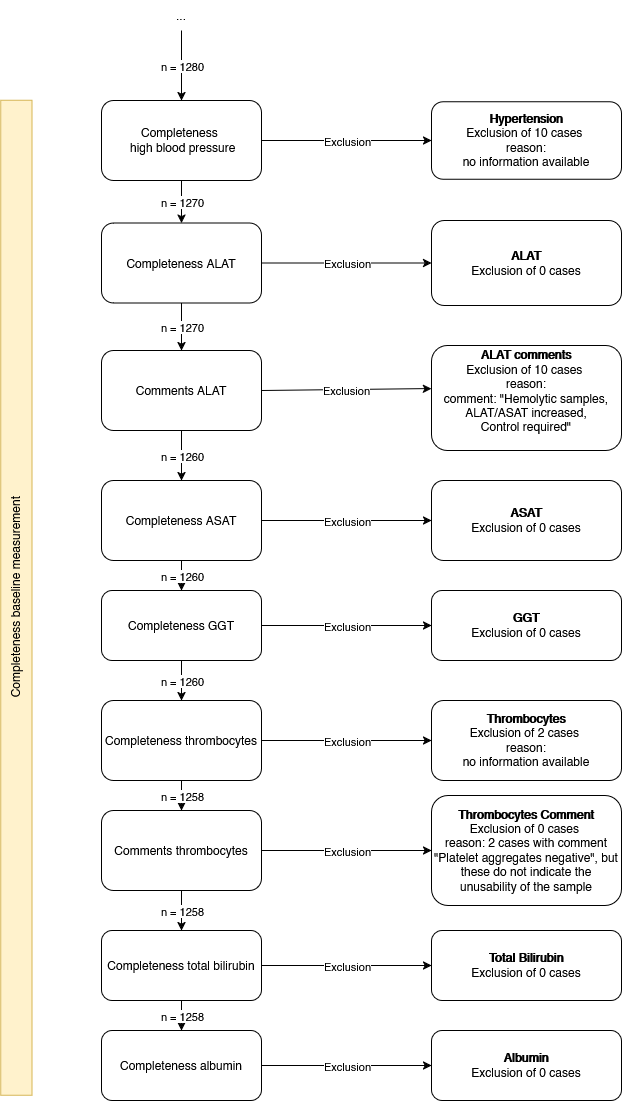
**

**Supplementary Figure 2** Data Selection II


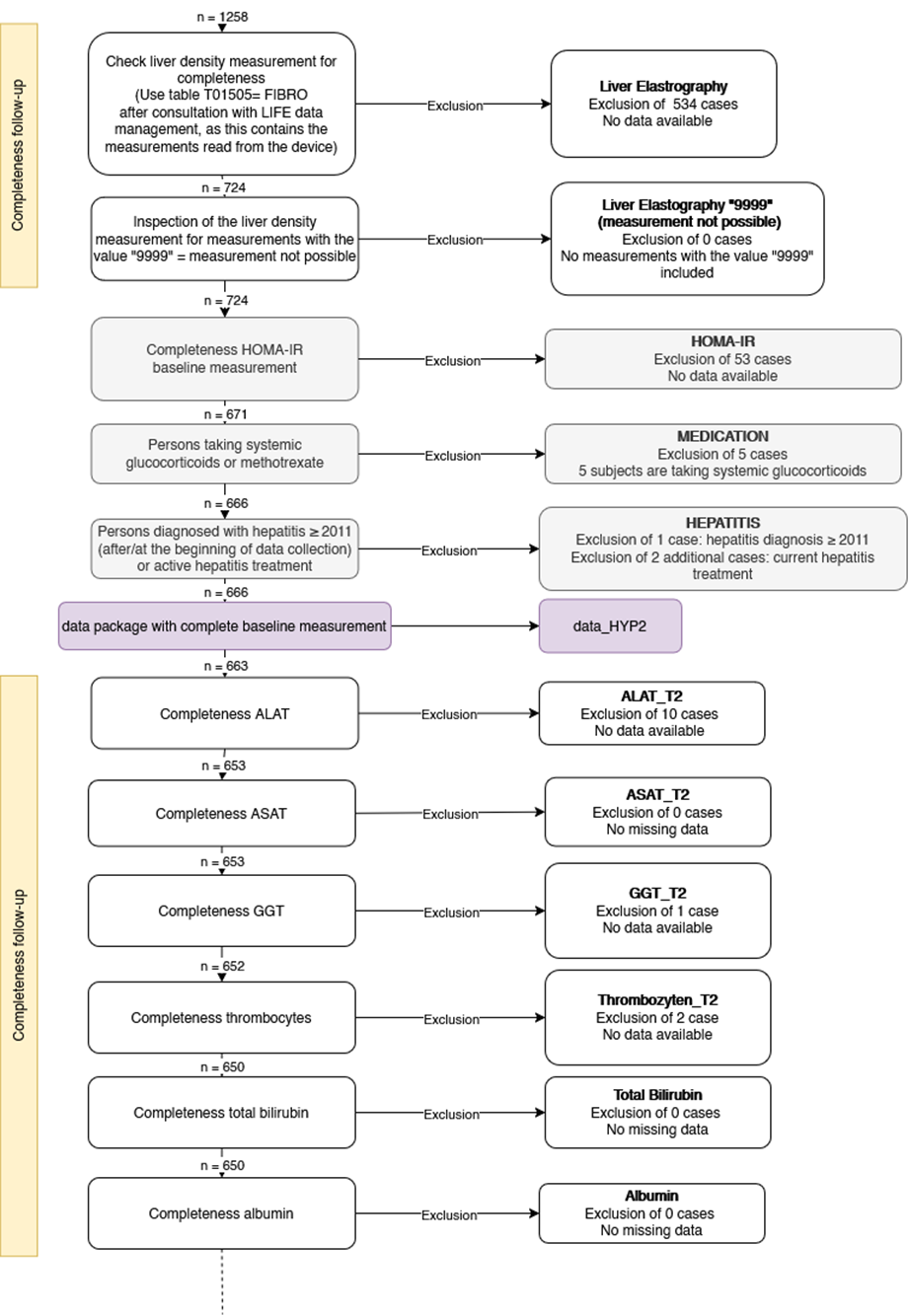


**Supplementary Figure 3** Data Selection III


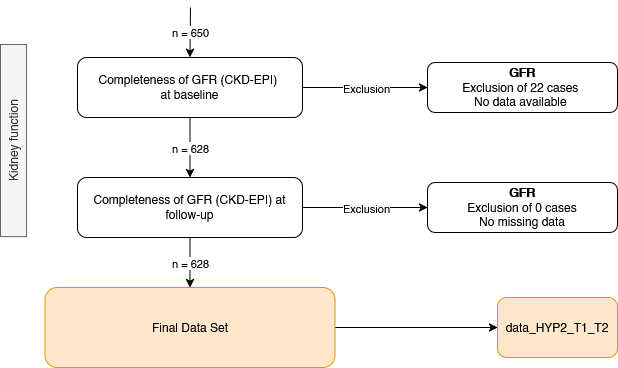


**Supplementary Figure 4** Data Selection IV

# Supplementary Table 1 categorial data of all subsets (liver elastography and BMI)

|  | Unstratified sample | | FIB > 1.3 (< 65 years) or  FIB >2.0 (≥ 65 years) | | BMI ≥ 30 | |
| --- | --- | --- | --- | --- | --- | --- |
|  | N | % | N | % | N | % |
| Male | 362 | 57.8 | 144 | 65.5 | 62 | 54.9 |
| Female | 264 | 42.2 | 76 | 35.5 | 51 | 45.1 |
| Never smoker | 359 | 57.4 | 120 | 54.6 | 51 | 45.1 |
| Former smoker | 194 | 31.0 | 87 | 39.6 | 51 | 45.1 |
| Active smoker | 73 | 11.6 | 13 | 5,8 | 11 | 9.8 |

#

| Alcohol intake | N | Min | Max | Mean | SD | Median | Male | Female |
| --- | --- | --- | --- | --- | --- | --- | --- | --- |
| 1 | 55 | 0.03 | 2.44 | 0.82 | 0.80 | 0.64 | 19 | 36 |
| 2 | 55 | 2.47 | 8.41 | 5.39 | 1.69 | 5.62 | 31 | 24 |
| 3 | 55 | 8.53 | 19.92 | 14.08 | 3.15 | 14.82 | 43 | 12 |
| 4 | 55 | 21.21 | 123.20 | 43.57 | 23.21 | 34.08 | 51 | 4 |
| HOMA-IR quantile | **N** | **Min** | **Max** | **Mean** | **SD** | **Median** | **Male** | **Female** |
| 1 | 29 | 0.42 | 2.24 | 1.64 | 0.46 | 1.66 | 12 | 17 |
| 2 | 28 | 2.27 | 3.38 | 2.86 | 0.34 | 2.86 | 14 | 14 |
| 3 | 28 | 3.38 | 4.93 | 4.11 | 0.50 | 4.05 | 16 | 12 |
| 4 | 28 | 5.04 | 65.77 | 9.45 | 11.46 | 6.33 | 20 | 8 |

# Supplementary Table 2 Descriptive data of alcohol quantiles and HOMA-IR quantiles
